# Association of Soluble Klotho Level with Adverse Outcomes in Patients on Maintenance Hemodialysis

**DOI:** 10.1155/2020/4923970

**Published:** 2020-11-12

**Authors:** Li-xia Yu, Qi-feng Liu, Jian-hua Feng, Sha-sha Li, Xiao-xia Gu, Yan Xiong, Qiang-Sun, Jian-Ming Ye

**Affiliations:** ^1^Department of Nephrology, Affiliated Kunshan Hospital of Jiangsu University, 91 Qianjin West Road, Kunshan, Jiangsu 215300, China; ^2^Clinical Research & Lab Centre, Affiliated Kunshan Hospital of Jiangsu University, 91 Qianjin West Road, Kunshan, Jiangsu 215300, China

## Abstract

**Background:**

The predictive value of soluble Klotho (sKlotho) for adverse outcomes in patients on maintenance hemodialysis (MHD) is controversial. In this study, we aimed to clarify the potential association of sKlotho levels with adverse outcomes in this patient population.

**Materials:**

A total of 211 patients on MHD were identified and stratified according to the median sKlotho level. Patients were followed up for adverse outcomes including cardiovascular (CV) morbidity and all-cause mortality.

**Results:**

During the 36-month follow-up, 75 patients [51 CV events (including 16 CV deaths) and 40 deaths] experienced adverse outcomes. After stratification according to median sKlotho level, patients with a lower sKlotho level had a greater risk of CV events (38.2% vs. 19.5%, *p* = 0.006), all-cause mortality (28.4% vs. 11.6%, *p* = 0.003), and combined adverse outcomes (51.0% vs. 24.2%, *p* < 0.001). Similar observations were made from analyses using Kaplan-Meier survival curves. Cox regression analysis showed that a low sKlotho level was strongly correlated with CV morbidity [1.942 (1.030–3.661), *p* = 0.040)], all-cause mortality [2.073 (1.023–4.203), *p* = 0.043], and combined adverse outcomes [1.818 (1.092–3.026), *p* = 0.021] in fully adjusted models.

**Conclusions:**

The sKlotho level was an independent predictive factor of adverse outcomes including CV morbidity and mortality in patients on MHD.

## 1. Introduction

The *Klotho* (*KL*) gene was first identified as an antiaging gene that encodes the Klotho protein in 1997 [1]. Klotho is a single-pass transmembrane protein that coworks with fibroblast growth factor (FGF)23 to modulate phosphorus and vitamin D metabolism [2]. The extracellular portion of Klotho can be cleaved by secretase, then enters the blood and urine [3]. This fragment is referred to as soluble or secreted Klotho (sKlotho). sKlotho has been found to confer pleiotropic cardiorenal benefits through reduction in oxidative stress and inflammation, inhibition of vascular and ectopic calcification and apoptosis, and amelioration of organ fibrosis [4–6]. These observations demonstrated that sKlotho acts as a novel protective factor against chronic kidney disease (CKD) and its complications, including cardiovascular (CV) morbidity.

The *KL* gene is strongly expressed in the kidneys. While it is also expressed in the thyroid gland, choroid, heart, aorta, and pituitary gland [7, 8], the kidneys are the primary source of sKlotho [7]. Consequently, sKlotho production declines when renal function is impaired [9]. Data from patients and animals with CKD and acute kidney injury consistently demonstrated systematic deficiency of sKlotho [10–12]. Notably, a reduced sKlotho level independently predicted an elevated risk of adverse renal outcomes or death for CKD patients not undergoing dialysis [11, 13]. These findings support the notion that sKlotho is associated with CKD progression and a novel prognostic biomarker associated with poor clinical outcomes in CKD patients.

Compared with CKD patients not undergoing dialysis, the sKlotho level in maintenance hemodialysis (MHD) patients was shown to be highly downregulated because of the complete loss of renal function [14]. Therefore, it is believed that reduced sKlotho level in MHD patients may be associated with additional adverse outcomes such as CV morbidity and mortality in this population. However, this association remains unclear. Several recent prospective studies have addressed the prognostic roles of sKlotho in the population on MHD, although the results were inconsistent [14–18]. Therefore, we designed the present prospective study to investigate the possible association between the sKlotho level and CV events and total mortality in patients on MHD to clarify the predictive significance of sKlotho in these patients.

## 2. Materials and Methods

### 2.1. Patients

This study was performed at the Blood Purification Center of Kunshan First People's Hospital. In total, 260 adult patients who underwent MHD for over 6 months were screened between August 1, 2016, and August 31, 2016. Exclusion criteria were listed as follows: age < 18 years old; evidence of cardiovascular events in the past 6 months; acute inflammation; underwent peritoneal dialysis; autoimmune diseases; history of malignancy, organ transplant, or immunosuppressant use; death within 3 months after enrollment; and refusal to cooperate with the study. All participants underwent bicarbonate hemodialysis at the frequency of three 4-hour sessions per week.

This study was conducted in agreement with the Declaration of Helsinki and approved by the Medical Ethics Committee of the Affiliated Kunshan Hospital of Jiangsu University. All patients were provided with informed consent.

### 2.2. Design

The design of this study is prospective. We first analyzed the baseline parameters of eligible participants via a cross-sectional analysis. Participants were then divided into two groups according to median sKlotho level (below or above median sKlotho level), and we proceeded to prospectively investigate whether decreased sKlotho level (below median sKlotho level) was associated with increased risk of total adverse outcomes including CV events and all-cause mortality. All patients were followed up for 36 months (from August 2016 to August 2019) or until the onset of CV events or death. The data was collected prospectively. The primary outcome was a CV event or all-cause mortality. CV events or morbidity were defined as new occurrences of cardiac insufficiency based on echocardiography or reduced ejection fraction, angina or myocardial infarction based on myocardial markers and electrocardiogram, or ischemic and hemorrhagic stroke based on magnetic resonance imaging or computed tomography scans. Regarding patients with recurrent or multiple episodes of CV events, the time of the initial onset was recorded for analyses. All-cause or total mortality was defined as death from any cause, and CV death was defined as death from any CV event. CV death was considered a severe CV event and was combined with CV events in the overall analysis.

### 2.3. Laboratory Tests

General medical data for each participant were recorded including age, sex, primary diseases, duration of MHD, dry body weight, and comorbid conditions. Routine biochemical parameters, including hemoglobin, blood urea nitrogen, serum creatinine (Scr), albumin (ALB), calcium, phosphate, potassium, and intact parathyroid hormone (iPTH), were assessed using standard laboratory techniques. KT/V was calculated using urea clearance (K), dialysis duration (T), and the patient's size (V). Fasting blood samples from all patients were obtained prior to hemodialysis. Samples were collected, centrifuged, separated, and stored at −80°C for additional use. Serum sKlotho and FGF23 levels in MHD patients were measured using a sandwich enzyme-linked immunosorbent assay (ELISA). The commercial ELISA kits for sKlotho and FGF23 were purchased from Santa Cruz Biotechnology.

### 2.4. Statistical Analysis

Normally distributed continuous data are presented as mean ± SD, and comparisons were made using a two-tailed Student's *t*-test. Other data are presented as median and interquartile range (25^th^ and 75^th^ percentiles), and comparisons were made using the Mann-Whitney *U* test. For categorical variables, data are presented as percentages, and comparisons were made using the chi-square test. For survival analysis, Kaplan-Meier curves were constructed to determine the possible relationship between the sKlotho level and cumulative adverse events using the log-rank test. Cox regression models (univariable or multivariable) were used to investigate potential risk factors associated with CV events or mortality. Results of Cox regression analyses are presented as hazard ratio (HR) and 95% confidence interval (CI). Data were analyzed using SPSS 19.0 software (SPSS, Inc., Chicago, IL, USA). Statistical significance was defined as *p* value < 0.05.

## 3. Results

### 3.1. Patient Characteristics

In total, 211 patients on MHD were enrolled and subjected to cross-sectional analysis based on the inclusion and exclusion criteria. Mean dialysis vintage was 9.18 ± 4.3 years and median sKlotho was 1.34 (range: 0.66–1.95) ng/mL, and then enrolled patients were divided into a low sKlotho group or a high sKlotho group according to this value (sKlotho < 1.34ng/mL or sKlotho ≥ 1.34ng/mL). Compared with patients in the above median sKlotho group, patients in the below median sKlotho group had lower use of vitamin D, lower Scr and iPTH levels, shorter hemodialysis durations, and higher FGF23 levels. Other parameters were compared between the two groups. Demographic and baseline characteristic parameters are shown in Table 1.

### 3.2. The Association between sKlotho Level and CV Morbidity and Mortality

**Figure 1:**
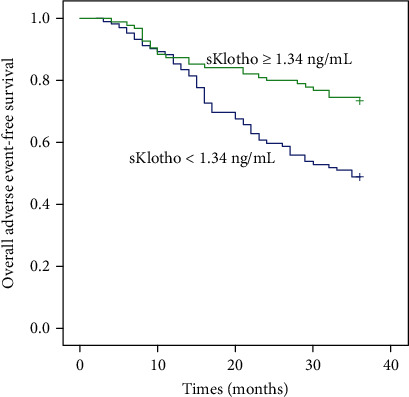
Kaplan-Meier curves of overall outcomes according to median sKlotho value (log-rank test, *p* = 0.001).

**Figure 2:**
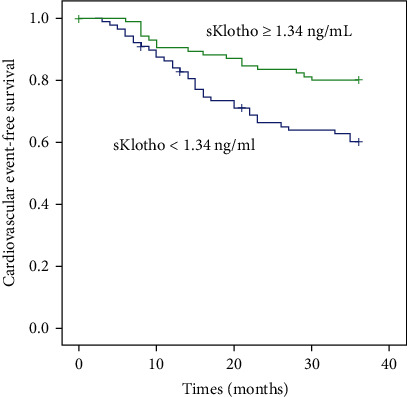
Kaplan-Meier curves of cardiovascular events according to median sKlotho value (log-rank test, *p* = 0.005).

**Figure 3:**
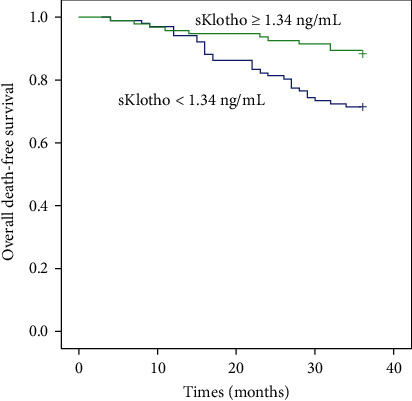
Kaplan-Meier curves of overall mortality according to median sKlotho value (log-rank test, *p* = 0.004).

Among the 211 participants, 11 had undergone renal transplantation and three were transferred to other dialysis centers and lost to follow-up. Therefore, 197 participants were eligible in the final prospective analysis. During the follow-up period of 36 months, 75 participants [51 CV events (including 16 CV deaths) and 40 deaths] experienced adverse outcomes. After stratifying by median sKlotho level, 52 patients in the low sKlotho group and 23 in the high sKlotho group experienced adverse outcomes, with significant difference (51.0% vs. 24.2%, *p* < 0.001). Even after stratifying by CV morbidity and mortality, more patients in the lower sKlotho group experienced CV events (38.2% vs. 19.5%, *p* = 0.006) and all-cause mortality (28.4% vs. 11.6%, *p* = 0.003) compared with the higher sKlotho group. Comparison of CV morbidity and all-cause mortality is shown in Table 2. Kaplan-Meier survival analysis suggested that the differences in the rates of overall adverse event-free survival (*p* = 0.001, Figure 1), CV event-free survival (*p* = 0.005, Figure 2), and all-cause mortality-free survival (*p* = 0.004, Figure 3) were significantly different (log-rank test) between the two groups.

### 3.3. Potential Risk Factors Correlated with CV Morbidity and Mortality

Cox regression was performed to clarify the potential risk factors correlated with CV morbidity and all-cause mortality. The results of univariate Cox regression analysis suggest that age, incidence of diabetes, dialysis vintage, ALB, and median sKlotho and FGF23 levels significantly affected total adverse events (Table 3). The risks of CV morbidity [2.235 (1.248–4.003), *p* = 0.007] and all-cause mortality [2.248 (1.393–3.625), *p* = 0.001] were both found to be >1.2-folds higher in the crude models. After adjustment for age, incidence of diabetes, dialysis vintage, ALB, and median FGF23 level, the median sKlotho level was still strongly associated with combined adverse outcomes [1.818 (1.092–3.026), *p* = 0.021] including CV morbidity [1.942 (1.030–3.661), *p* = 0.040)] and all-cause mortality [2.073 (1.023–4.203), *p* = 0.043] in the adjusted models (Table 4). Furthermore, if we defined sKlotho as a continuous variable, the similar association persists in the same adjusted model for combined adverse events [0.612 (0.423-0.886), *p* = 0.009)]. Dialysis vintage [0.971, (0.911-1.035), *p* = 0.971], diabetes [0.794, (0.487-1.296), *p* = 0.356], ALB [0.935, (0.872-1.001), *p* = 0.055], and FGF23 [0.821, (0.510-1.322), *p* = 0.418] were not associated with combined adverse outcomes in the adjusted models. Thus, our findings indicated that sKlotho level independently predicted the occurrence of adverse outcomes in MHD patients.

## 4. Discussion

In the current study, we presented that MHD patients with lower sKlotho levels experienced more adverse outcomes including CV events and all-cause mortality compared with those with higher sKlotho levels. Furthermore, lower sKlotho levels independently predicted adverse outcomes after adjusting for potential confounding factors. Our findings suggest circulating sKlotho represents a potential predictive factor for adverse outcomes in patients with MHD.

sKlotho is believed to be an active form of Klotho that is generated in the kidneys either by proteolytic cleavage of Klotho from the cell surface or through alternative mRNA splicing [19]. sKlotho functions as a secreted hormone and has pleiotropic protective effects on cells. Increasing evidence *in vivo* and *in vitro* has demonstrated that decreased sKlotho levels are related to the pathogenesis and development of CKD [12, 20–23]. More importantly, in longitudinal human studies, downregulated sKlotho levels were shown to correlate strongly with rapid decline of estimated glomerular filtration rate or increase in mortality in CKD patients not undergoing dialysis [13, 24]. All of the above studies support the notion that sKlotho may represent a novel potential prognostic biomarker for CKD patients [24–26]. This notion was also confirmed in our previous study [13].

The association between the sKlotho level and adverse clinical outcomes in MHD patients has been studied in recent years, although results have been inconsistent. Otani-Takei et al. first observed that a lower sKlotho level was independently associated with increased CV and total mortality rates [17]. Sixty-three patients on MHD were analyzed with a median follow-up of 65 months and a mean dialysis duration of 6.7 ± 5.4 years. After adjusting for potential confounders, the reduced sKlotho level independently predicted all-cause mortality (HR 6.33, 95% CI 1.70-25.44; *p* = 0.0065) [17]. Moreover, Marcais et al. recently demonstrated that an increased sKlotho level was associated with reduced occurrence of CV events and CV death (odds ratio, 0.39, CI 0.19–0.78, *p* = 0.008), and this trend persisted following adjustment for confounding factors [14]. In total, 238 MHD patients were followed up for 24 months, and median dialysis vintage was 3.6 years in this study [14]. Consistent with the previous reports, we found that patients with sKlotho levels above the median value had fewer adverse outcomes (CV events and all-cause mortality). Moreover, the association persisted even in the fully adjusted models. Interestingly, we found that patients enrolled in our study had the longest dialysis vintage, which was 9.18 ± 4.30 years. After adjustment for dialysis vintage and other confounders, sKlotho was still associated with adverse outcomes. The impact of sKlotho was not attenuated by longer dialysis vintage. This is distinguished from other studies with short hemodialysis durations [14, 15, 17]. Taken together, these findings strongly suggest that sKlotho has prognostic value in MHD patients, especially for ones with long dialysis vintage.

Several potential mechanisms may explain this phenomenon. First, sKlotho was recently shown to have a novel role in maintaining calcium-phosphate homeostasis via FGF23-dependent and FGF23-independent mechanisms [2]. A decreased sKlotho level was shown to be accompanied with calcium-phosphate metabolic disorders, which was independently associated with increased CV events and risk of mortality in MHD patients [27]. Second, sKlotho emerged as an inhibitory factor for vascular calcification (VC). VC is a known risk factor affecting adverse outcomes in MHD patients [28–30]. Klotho is also expressed in vascular tissue, and Klotho deficiency in mice caused severe calcification in vascular and soft tissues [31]. In clinical studies, lower sKlotho levels were shown to be closely linked to increased artery calcification, coronary artery calcification, and valve calcification in dialysis patients [32–34]. Calcification in vascular or soft tissue can most certainly greatly increase detrimental outcomes in MHD patients. Third, a recent study showed that low sKlotho levels correlated with the occurrence of atrial fibrillation in MHD patients. Atrial fibrillation is a known and important risk factor associated with CV events and mortality. Finally, sKlotho exerts protective effects against inflammatory and oxidative stress [4, 35], which increase with age [36, 37]. sKlotho has been shown to inhibit inflammation and oxidative stress via several mechanisms [38–40]. A reduced sKlotho level was shown to be accompanied by an enhanced inflammatory response and oxidative stress, which were potentially linked to poor survival in patients on MHD.

However, other studies yielded contrasting results, showing that the sKlotho level was not a predictor in MHD patients. A recent study by Zheng et al. showed that compared with MHD patients with high sKlotho levels, patients with low sKlotho levels had shorter survival time and increased risk of mortality, although the correlation disappeared after adjustment for age and diabetes [34]. The study included 128 participants with MHD and follow-up duration of 3 years, but a significant difference in baseline parameters [such as age (61.9 ± 15.4 vs. 56.2 ± 12.6, *p* < 0.001) and prevalence of diabetes (37.5% vs. 23.3%, *p* = 0.005)] was observed. Nowak et al. also demonstrated that FGF23, rather than Klotho, was independently associated with all-cause mortality in crude and adjusted models analyzing 239 MHD patients during a median follow-up interval of 2.5 years [16]. Buiten et al. found that MHD patients with low sKlotho levels were prone to experience more CV events, although the relationship was not observed in multivariate regression models following multiple adjustments [15]. The study was cross-sectional, with a relatively small sample size, and no follow-up was conducted [15]. The discrepancies in observations among these studies may be explained by differences in study design or differences in baseline parameters of the study participants such as age, sex, region of origin, dialysis modality, dialysis vintage, and biochemical parameters. Variations in sKlotho commercial assays may also explain the inconsistencies [41]. Therefore, these conflicting results should be carefully interpreted.

The present study had several limitations. First, vascular and soft tissue calcification was not assessed in our patients. Therefore, a direct association between the sKlotho level and VC was not shown, and an accurate evaluation of the independent effects of VC on CV events or mortality was not performed. Second, the states of inflammation and oxidative stress, which influence the sKlotho level and outcomes [40, 42], were not assessed between the two groups. Third, the follow-up period of this study was relatively short, with a relatively small sample size. Fourth, repeated blood samples were not taken, and the possible intraindividual variations of sKlotho levels were not excluded. Moreover, we only included a small group of MHD patients with long dialysis vintage and excluded patients who underwent predialysis, incident dialysis, or peritoneal dialysis. Because of the aforementioned limitations, our results may be over- or underestimated. Therefore, our finding may not apply to the entire population of CKD patients.

In conclusion, the decreased sKlotho level was associated with more occurrences of adverse outcomes. We provide evidence that sKlotho represents a potential prognostic biomarker for patients with MHD, especially for long-term dialysis vintage. Additional well-designed studies are required to further validate our findings.

## Figures and Tables

**Table 1:** Comparison of demographic and clinical data in enrolled patients on MHD.

Variables	Total (*n* = 211)	Klotho < 1.34 ng/ml (*n* = 105)	Klotho ≥ 1.34 ng/ml (*n* = 106)	*p* value
Ages	56.2 ± 12.6	57.1 ± 13.2	55.3 ± 12.0	0.283
Male (%)	116 (55.0)	55 (52.4)	61 (57.5)	0.451
Diabetes (%)	16249 (23.3)	23 (21.9)	26 (24.5)	0.652
Hypertension (%)	179 (84.8)	88 (83.1)	91 (85.5)	0.680
ACEI/ARB (%)	168 (79.6)	82 (78.1)	86 (81.1)	0.584
Vitamin D (%)	176 (83.6)	81 (77.1)	95 (89.6)	0.015
Dialysis vintage (year)	9.18 ± 4.30	8.36 ± 3.50	10.0 ± 4.85	0.005
Scr (*μ*mol/L)	1057.1 ± 265.5	1013.0 ± 251.6	1101.0 ± 272.7	0.016
Bun (mmol/L)	24.1 ± 5.7	24.2 ± 6.3	24.1 ± 5.1	0.837
KT/V (per week)	1.64 ± 0.65	1.66 ± 0.62	1.62 ± 0.66	0.682
HGB (g/L)	107.8 ± 15.5	106.4 ± 16.6	109.1 ± 14.3	0.337
Ca (mmol/L)	2.16 ± 0.22	2.18 ± 0.25	2.13 ± 0.18	0.136
P (mmol/L)	1.86 ± 0.59	1.87 ± 0.62	1.86 ± 0.57	0.942
Ca × P (mg^2^/dL^2^)	4.04 ± 1.41	4.08 ± 1.47	4.00 ± 1.34	0.667
ALB (g/L)	40.0 ± 3.5	39.8 ± 3.6	40.3 ± 3.3	0.337
iPTH (pg/mL)	285.2 (137.2, 568.4)	273.4 (126.4, 572.3)	316.5 (163.0, 561.3)	0.530
Klotho (ng/mL)	1.34 (0.66, 1.95)	0.66 (0.39, 0.93)	1.95 (1.61, 2.26)	<0.001
FGF23 (pg/mL)	515.3 (401.0, 661.4)	582.6 (450.6, 698.3)	482.8 (352.5, 598.6)	0.007

Abbreviations: MHD: maintenance hemodialysis; Scr: serum creatinine; Ca: calcium; P: phosphorus; Bun: blood urea nitrogen; ALB: albumin; HGB: hemoglobin; ACEI: angiotensin-converting enzyme inhibitors; ARB: angiotensin receptor blockers; iPTH: intact parathyroid hormone; FGF23: fibroblast growth factor 23. Data are presented as mean ± standarddeviation for normally distributed variables, otherwise median with 25^th^-75th percentile.

**Table 2:** Clinical outcomes according to median Klotho level.

Outcomes	Klotho < 1.34ng/mL (*n* = 102, %)	Klotho ≥ 1.34ng/mL (*n* = 95, %)	*p* value
All-cause mortality	29 (28.4%)	11 (11.6%)	0.003
Cardiovascular	11 (11.0%)	5 (5.2%)	/
Infection	6 (6.0%)	3 (3.1%)	/
Tumor	3 (3.0%)	1 (1.0%)	/
Others	9 (9.0%)	2 (2.1%)	/
Cardiovascular morbidity	34/89 (38.2%)	17/87 (19.5%)	0.006
Composite endpoints	52 (51.0%)	23 (24.2)	<0.001

**Table 3:** Univariate Cox regression analysis for combined adverse events.

Parameters	HR (95% CI)	*p* value
Univariate analysis		
Age	1.047 (1.026-1.068)	<0.001
Gender (male/female)	1.463 (0.935-2.289)	0.096
Hypertension (yes/no)	1.155 (0.624-2.137)	0.647
Diabetes (yes/no)	0.583 (0.363-0.935)	0.025
Vitamin D (yes/no)	1.524 (0.892-2.605)	0.123
ACEI/ARB (yes/no)	1.274 (0.743-2.183)	0.378
Dialysis vintage (year)	0.930 (0.872-0.992)	0.027
KT/V (per week)	0.903 (0.624-1.307)	0.588
Ca × P (mg^2^/dL^2^)	0.854 (0.722-1.010)	0.066
HGB (g/L)	0.995 (0.981-1.010)	0.495
ALB (g/L)	0.903 (0.846-0.965)	0.002
Klotho (median)	2.248 (1.393-3.625)	0.001
FGF23 (median)	0.595 (0.377-0.943)	0.027
iPTH (ng/mL)	0.999 (0.999-1.000)	0.080

**Table 4:** Multivariate Cox regression for cardiovascular events and combined adverse events.

Models	Cardiovascular events		Combined adverse events	
	HR (95% CI)	*p* value	HR (95% CI)	*p* value
Model 1	2.235 (1.248-4.003)	0.007	2.248 (1.393-3.625)	0.001
Model 2	2.201 (1.228-3.944)	0.008	2.072 (1.279-3.356)	0.003
Model 3	1.986 (1.066-3.701)	0.031	1.824 (1.106-3.010)	0.019
Model 4	1.942 (1.030-3.661)	0.040	1.818 (1.092-3.026)	0.021

Model 1: crude; Model 2: adjustment for age; Model 3: Model 2 plus adjustment for diabetes and dialysis vintage; Model 4: Model3 plus adjustment for albumin and FGF23.

## Data Availability

All data can be shared freely upon reasonable request.
